# Advancements in Early Detection and Screening Strategies for Pancreatic Cancer: From Genetic Susceptibility to Novel Biomarkers

**DOI:** 10.3390/jcm13164706

**Published:** 2024-08-10

**Authors:** Yash Shah, Dushyant Singh Dahiya, Angad Tiwari, Harendra Kumar, Manesh Kumar Gangwani, Hassam Ali, Umar Hayat, Saqr Alsakarneh, Sahib Singh, Sheza Malik, Amir H. Sohail, Saurabh Chandan, Meer A. Ali, Sumant Inamdar

**Affiliations:** 1 Department of Internal Medicine, Trinity Health Oakland/Wayne State University, Pontiac, MI 48341, USA; 2 Division of Gastroenterology, Hepatology & Motility, The University of Kansas School of Medicine, Kansas City, KS 66160, USA; 3 Department of Internal Medicine, Maharani Laxmi Bai Medical College, Jhansi 284001, Uttar Pradesh, India; 4 Department of Internal Medicine, Dow University of Health Sciences, Karachi 74200, Pakistan; 5 Department of Gastroenterology and Hepatology, University of Arkansas For Medical Sciences, Little Rock, AR 72205, USA; 6 Division of Gastroenterology, Hepatology & Nutrition, East Carolina University/Brody School of Medicine, Greenville, NC 27834, USA; 7 Department of Internal Medicine, Geisinger Wyoming Valley Medical Center, Wilkes Barre, PA 18711, USA; 8 Department of Internal Medicine, University of Missouri-Kansas City, Kansas City, MO 64110, USA; 9 Department of Internal Medicine, Sinai Hospital, Baltimore, MD 21215, USA; 10 Department of Internal Medicine, Rochester General Hospital, Rochester, NY 14621, USA; 11 Department of Surgery, University of New Mexico, Albuquerque, NM 87131, USA; 12 Center for Interventional Endoscopy (CIE), Advent Health, Orlando, FL 32803, USA

**Keywords:** pancreatic cancer, screening, high risk, hereditary

## Abstract

Pancreatic cancer is a rare but lethal cancer due to its biologically aggressive nature, advanced stage at the time of diagnosis, and poor response to oncologic therapies. The risk of pancreatic cancer is significantly higher to 5% in certain high-risk individuals with inherited genetic susceptibility. Screening for pancreatic cancer in these individuals from high-risk groups can help with the early detection of pancreatic cancer as well as the detection of precursor lesions leading to early surgical resection and improved overall outcomes. The advancements in radiological imaging as well as advanced endoscopic procedures has made a significant impact on the early diagnosis, surveillance, and staging of pancreatic cancer. There is also a significant advancement in the development of biomarkers for the early detection of pancreatic cancer, which has also led to the development of liquid biopsy, allowing for microRNA detection in serum and circulating tumor cells. Various societies and organizations have provided guidelines for pancreatic cancer screening and surveillance in high-risk individuals. In this review, we aim to discuss the hereditary risk factors for developing pancreatic cancer, summarize the screening recommendations by different societies, and discuss the development of novel biomarkers and areas for future research in pancreatic cancer screening for high-risk individuals.

## 1. Introduction

Pancreatic cancer is the third leading cause of cancer-related deaths in the United States after lung and colon cancer, with an estimated 66,440 new cases of pancreatic cancer and 51,570 pancreatic-cancer-related deaths in 2024 as per the American Cancer Society [1,2]. The rate of new cases and deaths of pancreatic cancer is 13.3 and 11.1, respectively, per 100,000 men and women per year based on the 2016–2020 cases and deaths [3]. In 2020, approximately 95,389 people were living with pancreatic cancer in the United States [3]. The extensive presence of over 4312 completed or ongoing clinical trials as gathered from the clinicaltrials.gov database in April 2024 depicts the gravity of the situation. Pancreatic cancer is associated with a low survival rate, and the 5-year relative survival of patients with pancreatic cancer from 2013 to 2019 was 12.5% based on the National Cancer Institute [3]. There has been no significant change in the overall survival rate of patients with pancreatic cancer over the years despite the development of and improvement in the understanding of pancreatic cancer and surgical techniques, as the tumor is already unresectable in 80% of symptomatic patients at the time of diagnosis [4]. There is a significant difference in the 5-year relative survival rate between the pancreatic stages, with 42% survival in patients at the localized stage, 14% in patients at the regional stage, and 3% in patients with distant metastasis [5,6]. Therefore, early pancreatic cancer detection is crucial and can lead to better patient outcomes. 

Pancreatic cancer develops in three possible settings, which include sporadic in about 90% of the patients, familial pancreatic cancer in 7% of cases, and inherited cancer syndromes in about 3% of patients [7,8]. The risk factors for pancreatic cancer can be classified into genetic and non-genetic as well as modifiable and non-modifiable [9]. One of the modifiable risk factors associated with an increased risk of pancreatic cancer is tobacco smoking, which increases carcinogenesis by inducing inflammatory cells, generating fibrosis, inhibiting apoptosis, and eventually the proliferation of pancreatic cells [10,11]. Other modifiable risk factors include diabetes mellitus, heavy alcohol consumption (24 g/dL), chronic pancreatitis, and obesity [12,13,14,15].

Several genetic syndromes have also been linked to an increased risk of pancreatic cancer. The American Gastroenterological Association (AGA) has defined familial pancreatic cancer (FPC) as pancreatic cancer occurring in two or more first-degree relatives that do not meet the criteria for other hereditary cancer syndromes [16]. Some of the genetic syndromes and the mutations associated with an increased risk of pancreatic cancer are Peutz–Jeghers syndrome associated with the *STK11* gene, hereditary pancreatitis associated with the *PRSS1, SPINK1, CTRC,* and *CFTR* genes, familial atypical multiple mole melanoma associated with the *CDKN2A* gene, Li–Fraumeni syndrome associated with the *TP53* gene, Lynch syndrome associated with the *MLH1, MSH2, MSG6*, and *PMS2* genes, hereditary breast–ovarian cancer syndrome associated with the *BRCA1* and *BRCA2* genes, familial adenomatous polyposis associated with the *APC* and *MUTYH* genes, and ataxia telangiectasia associated with an *ATM* gene mutation [7,9]. Appropriate screening protocols are needed for the early diagnosis and management of pancreatic cancer in these patients from high-risk populations. In this review, we will discuss the high-risk population for pancreatic cancer and the approach to pancreatic screening in these patients, along with different available modalities and future directions.

## 2. Risk Factors for Pancreatic Cancer

### 2.1. Genetic and Familial Risk Factors 

Several important pancreatic cancer susceptibility genes have been identified due to the recent advancement in genome sequencing and array genotyping. There is a wide spectrum of genetic variation with high-penetrance genes leading to a very high lifetime risk of developing diseases as compared to low-penetrance genes, which are encountered more commonly but have a minor or modest risk of disease [15]. Individuals with a history of pancreatic cancer in a first-degree relative have an increased risk of developing pancreatic cancer (OR 2.1–5.3 and RR 1.5–1.7) [17,18]. The incidence of pancreatic cancer is also dependent on the number of first-degree relatives with a history of pancreatic cancer, and the standardized incidence ratio (SIR) of pancreatic cancer in an individual with one first-degree relative with pancreatic cancer is 4.5, 6.4 for two first-degree relatives with pancreatic cancer, and ≥32 for three first-degree relatives with pancreatic cancer [19]. It is important to know that patients with non-genetic syndromes are excluded from the diagnosis of hereditary pancreatic cancer. A risk prediction tool for pancreatic cancer has been developed by Wang et al., which is known as PancPRO, and it can help to quantify an individual’s risk of developing pancreatic cancer based on the family history [20].

#### 2.1.1. Peutz–Jeghers Syndrome

Peutz–Jeghers syndrome is an autosomal dominant inherited disorder that is associated with the mutation of the *STK11* gene on chromosome 19p13, which normally acts as a tumor suppressor gene [21,22]. It has been associated with the development of several tumors including esophageal, stomach, small intestine, colon, lung, breast, uterine, ovarian, and pancreatic [23]. A meta-analysis by Giardiello et al. in 2000 showed that pancreatic cancer was noted in 6 out of 66 individuals with Peutz–Jeghers syndrome, with a relative risk of 132 and a rate of 118.6 per 100,000 person-years [23]. A study on 144 patients with Peutz–Jeghers syndrome showed that 7 patients developed pancreatic cancer with a cumulative risk of 2.4% at age 40, 3.9% at age 50, 11.1% at age 60, and 25.6% at age 70 years [22]. Another study by Resta et al. had similar results with a relative risk of 139.7 and a cumulative risk of 2–55% of developing pancreatic cancer in patients with Peutz–Jeghers syndrome associated with a *STK11* gene mutation [24].

#### 2.1.2. Lynch Syndrome

Lynch syndrome, which is also known as hereditary non-polyposis colorectal cancer, is an autosomal dominant condition caused by defects in mismatch repair genes like *MLH1, MSH2, MSH6*, or *PMS2*, and associated with increased risk of colorectal cancer, endometrial cancer, and pancreatic cancer. A study by Kastrinos et al. on 147 families with mutations in *MLH1, MSH2*, or *MSH6* genes showed an 8.6-fold increase in the risk of pancreatic cancer as compared to the general population, and the estimated cumulative risk of developing pancreatic cancer was 1.3% up to the age of 50 years and 3.7% up to the age of 70 years [25]. Another study by Borelli et al. showed that a *MLH1* (c.2252_2253delAA) mutation in patients with Lynch syndrome is associated with an increased risk of pancreatic tumors, with 5 patients having pancreatic cancer out of a total of 67 patients with Lynch syndrome [26]. The relative risk of pancreatic cancer in patients with Lynch syndrome is 5–9 [9].

#### 2.1.3. Hereditary Breast and Ovarian Cancer

Hereditary breast and ovarian cancer is an autosomal dominant disorder characterized by the mutation of *BRCA1* and *BRCA2* genes, which leads to an increased risk of breast cancer, ovarian cancer, and to a lesser extent, prostate cancer, pancreatic cancer, and melanoma, especially in patients with a *BRCA2* mutation [27]. Most patients with hereditary breast and ovarian cancer have triple-negative breast cancer (48–66%) [28,29]. The risk of pancreatic cancer is 1–3% in patients with a BRCA1 mutation and 3–5% by the age of 70 years in patients with a *BRCA2* mutation as compared to the general population with a risk of 0.5% [27,30]. However, a study by Olakowski et al. showed that pancreatic cancer is the third most common in patients with hereditary breast and ovarian syndrome [31]. A multi-center study by Thompson et al. showed that the relative risk of pancreatic cancer in patients with a *BRCA1* mutation is 2.26 (95% CI: 1.26–4.06), and another study by Easton et al. showed that the relative risk of pancreatic cancer in patients with a *BRCA2* mutation is 3.51 (95% CI: 1.87–6.58) [32,33]. *PALB2* is a partner and localizer of the *BRCA2* gene that participates in DNA repair by binding and colocalizing with the *BRCA2* gene [34]. Mutations in the *PALB2* gene have been associated with an increased risk of familial pancreatic cancer, with an overall prevalence of 3.1% in a study conducted by Jones et al. and 3.7% in a study by Slater et al. [35,36].

#### 2.1.4. Familial Atypical Multiple Mole Melanoma (FAMMM)

Familial atypical multiple mole melanoma (FAMMM) is an autosomal dominant genodermatosis characterized by a mutation in the *CDKN2A* gene, which is characterized by multiple melanocytic nevi and also an increased risk of pancreatic cancer. A study of the world’s largest melanoma database showed a significant association between pancreatic cancer and *CDKN2A* mutation in melanoma multiplex families, with 28% of mutation-positive kindreds having pancreatic cancer as compared to 6% in patients without the mutation [37]. A study by Vasen et al. on 27 families showed that pancreatic cancer was the second most common in patients with familial atypical multiple mole melanoma, with an estimated cumulative risk of 17% by the age of 75 years [38].

#### 2.1.5. Li–Fraumeni Syndrome (LFS)

Li–Fraumeni syndrome (LFS) is an autosomal dominant disorder due to germline mutations in the *TP53* gene [39,40,41]. The diagnostic criteria for classic LFS include an individual with sarcoma before age 45 years, another first- or second-degree relative with any cancer before age 45 years, and another first- or second-degree relative with sarcoma diagnosed at any age or another cancer diagnosed before age 45 years [42]. The International Agency for Research on Cancer (IARC) germline *TP53* database showed that pancreatic cancer occurred in 1.2% of the affected individuals with a median age of 53 years at the time of diagnosis [43]. The relative risk of pancreatic cancer in patients with LFS is 7.73 (95% CI: 2–19) as compared to the population without LFS [44,45].

#### 2.1.6. Familial Adenomatous Polyposis (FAP)

Familial adenomatous polyposis (FAP) is an autosomal dominant disorder due to the germline mutation in the *APC* gene located on chromosome 5q21 and it affects one in 10,000–20,000 people [46,47]. The patients with FAP have a four-fold higher risk of pancreatic cancer as compared to the general population with a total risk of around 2% over their lifetime [48]. A case–control study of 565 patients with FAP and 1080 controls in a Danish population showed that the hazard ratio for developing pancreatic cancer in patients with FAP is 6.45 (95% CI: 2.02–20.64; *p* = 0.002) as compared to the normal population [49]. Another study by Giardiello et al. showed that the relative risk of pancreatic adenocarcinoma in patients with FAP is 4.46 (95% CI: 1.2–11.4) [50]. Some atypical pancreatic tumors like pseudopapillary tumor, acinar cell carcinoma, pancreatoblastoma, and neuroendocrine tumor (NET) are also associated with FAP [46,51].

#### 2.1.7. Ataxia–Telangiectasia Mutated (ATM)

The *ataxia–telangiectasia mutated* (*ATM*) gene is a serine/threonine kinase that plays an important role in the repair of DNA double-strand break repair, control, and apoptosis [52,53]. The mutation in the *ATM* gene is associated with an increased risk of breast cancer; however, the data on the risk of pancreatic cancer in individuals with kindreds of pathogenic variations are limited [54]. A study by Hall et al. showed an increased risk (OR 4.21, 95% CI: 3.24–5.47) of pancreatic cancer in patients with an *ATM* gene mutation [55]. A multi-center cohort study of 2227 individuals of 130 pancreatic cancer kindreds by Hsu et al. found that the risk of pancreatic cancer in patients with a pathogenic variation of the *ATM* gene is 1.1% by age 50 years, 6.3% by age 70 years, and 9.5% by age 80 years [54]. The overall relative risk of pancreatic cancer in patients with pathogenic *ATM* variants is 6.5 (95% CI: 4.5–9.5) [54]. A study by Geoffrey-Perez et al. did not show any significant difference in the risk of developing pancreatic cancer in individuals with pathogenic variants of the *ATM* gene [56]. Another study by Hannan et al. showed that the *ATM* mutation may be prognostic for improved outcomes in patients with pancreatic ductal adenocarcinoma, with overall survival of 40.2 months as compared to 15.5 months in patients without a pathogenic variant of *ATM* (HR 0.14, 95% CI: 0.04–0.47) [57].

#### 2.1.8. Cystic Fibrosis

Cystic fibrosis is a monogenic disease due to the mutation of the *cystic fibrosis transmembrane conductance regulator (CFTR)* gene, affecting the lungs as well as extrapulmonary tissues including the gastrointestinal tract [58,59]. Cystic fibrosis leads to pancreatic insufficiency due to pancreatic damage, the loss of acinar cell fatty replacement, as well as interstitial fibrosis, which eventually leads to chronic inflammatory pancreatitis [59]. Chronic pancreatitis has been associated with an increased risk of pancreatitis, and a study by Maisonneuve et al. showed that the risk ratio for pancreatic cancer in patients with a *CFTR* gene mutation is 5.3 (95% CI: 2.4–10.1) as compared to individuals without a *CFTR* gene mutation [60]. A case–control study by McWilliams et al. has also shown higher odds of developing pancreatic cancer in patients with a *CFTR* gene mutation as compared to controls (OR 1.40, 95% CI: 1.04–1.89), with a higher carrier frequency in younger (<60 years) individuals (OR 1.82, 95% CI: 1.14–2.94) [61]. The relative risk of pancreatic cancer in patients with a *CFTR* gene mutation is 5.3 (95% CI: 2.4–10.1) [44,60,62].

#### 2.1.9. Hereditary Pancreatitis

Hereditary pancreatitis is an autosomal dominant disorder and a rare cause of chronic pancreatitis due to a genetic defect in the sequencing analysis of the 7q35 chromosome region of the *PRSS1* gene [63]. Recurrent or persistent chronic inflammation is considered an important risk factor for cancer development in patients with chronic pancreatitis [64]. A multi-center study by Lowenfels et al. on 246 patients with hereditary pancreatitis showed that the standardized incidence ratio of pancreatic cancer in patients with hereditary pancreatitis is 53 (95% CI: 23–105), and the estimated cumulative risk of developing pancreatic cancer by age 70 years is 40%, with a remarkably higher risk of 75% in patients with a paternal inheritance pattern [65]. The risk of pancreatic cancer associated with the different hereditary conditions discussed above is summarized in Table 1.

### 2.2. Non-Genetic and Environmental Risk Factors

In addition to the generic and familial risk factors, some of the most common environmental and non-genetic risk factors associated with an increased risk of pancreatic cancer are tobacco use, alcohol consumption, chronic pancreatitis, obesity, and diabetes mellitus [66]. Most of these conditions lead to chronic inflammation and subsequently an imbalance in oncogenic–anti-oncogenic milieu. Tobacco use can lead to chronic inflammation and pathogenic mutation in the *KRAS* and *p53* genes, leading to cytokine induction and growth factor stimulation, and is responsible for 20–35% of cases of pancreatic cancer [67]. Similarly, heavy alcohol consumption can also lead to an increased risk of pancreatic cancer by 1.22–1.36, as alcohol and its metabolites are pro-carcinogenic and lead to cellular gene instability [68]. Chronic pancreatitis can lead to the increased production of inflammatory cytokines like TNFα, IL-6, IL-8, PDGF, and TGFβ, which leads to the increased production of reactive oxygen species, cellular proliferation, and cellular transformation [69]. A study by Raimondi et al. showed that chronic pancreatitis has a relative risk of 13.2 for pancreatic cancer [70]. Obesity can lead to adiposopathy, which is a pro-inflammatory condition leading to dysregulation in the hormone levels of leptin and adiponectin, and leads to an increased risk of pancreatic cancer by 1.12 for each increase in body metabolic index by 5 kg/m^2^ [71,72]. The association between pancreatic cancer and diabetes mellitus is unclear as about 80% of people with pancreatic cancer have glucose intolerance, supporting that diabetes can be a consequence of pancreatic cancer [73]. However, various studies have shown that diabetes mellitus can lead to an increased risk of pancreatic cancer due to increased levels of insulin and insulin-like growth factor 1, inducing pancreatic glandular proliferation as well as the interaction between pancreatic stellate cells and tumor-associated macrophages [74,75]. This interaction leads to fibrosis, cellular proliferation, and apoptosis inhibition, leading to an increased risk of pancreatic cancer [75].

## 3. Age-Appropriate Screening for Pancreatic Cancer in High-Risk Groups

The mean age for the diagnosis of pancreatic cancer in individuals with familial pancreatic cancer is 68 years with an increasing number of lesions in individuals older than 50 years and an increase in lesions with high-grade dysplasia in those older than 65 years [76]. The American Gastroenterology Association (AGA) recommends the initiation of pancreatic cancer screening in individuals of high-risk group at the age of 50 years or 10 years younger than the initial age of onset [16]. The International Cancer for the Pancreas Screening (CAPS) Consortium has similar guidelines for beginning surveillance at the age of 50 years or 10 years younger than the youngest relative with pancreatic cancer (Grade 2: probably do it) or at the age of 55 years or 10 years younger than the youngest relative with pancreatic cancer (Grade 2: probably do it) [77]. The recommendation to start screening at the age of 45 years or 10 years younger than the youngest relative with pancreatic cancer is Grade 4 (probably do not do it) [77]. The recommendation for surveillance by CAPS for patients with Peutz–Jeghers syndrome is 40 years or 10 years younger than the youngest relative with pancreatic cancer (Grade 2: probably do it), and it is Grade 4 (probably do not do it) for beginning surveillance at 30 or 35 years [77]. The AGA recommends screening starting at age 35 years in patients with Peutz–Jeghers Syndrome due to the younger age of onset of pancreatic cancer in these patients, which aligns with the recommendations by the ASGE [16,78]. The AGA recommends initiating surveillance for pancreatic cancer in patients with a PRSS1 mutation and a CDKN2A mutation at the age of 40 years, which is similar to that by CAPS and the ASGE [16,77,78]. CAPS recommends pancreatic cancer screening beginning at the age of 50 years or 5 years earlier than for high-risk individuals with defined familial pancreatic cancer in patients with BRCA2, ATM, and PALB2 mutations (Grade 2: probably do it) [77]. The American Society of Gastrointestinal Endoscopy (ASGE) has similar recommendations for patients with BRCA1, BRCA1, and PALB2 mutations to begin screening for pancreatic cancer at the age of 50 years or 10 years younger than the youngest relative with pancreatic cancer [78]. Surveillance is recommended starting at the age of 50 years or 10 years younger than the youngest relative with pancreatic cancer in patients with Lynch syndrome by the AGA and ASGE [16,78]. CAPS recommends pancreatic cancer screening 5 years earlier in all individuals with germline mutation (except Peutz–Jeghers syndrome) as compared to high-risk individuals without germline mutations (Grade 2: probably do it) [77]. The National Comprehensive Cancer Network (NCCN) recommends screening for pancreatic cancer starting at age 50 or younger depending on the family history in patients with a TP53 mutation [79]. The ASGE recommends screening for pancreatic cancer starting at age 50 or in patients with an ATM mutation and first- or second-degree relatives with a pancreatic cancer history 10 years younger than the youngest relative with pancreatic cancer, which is similar to the AGA and CAPS [16,77,78]. The recommendations for the screening of pancreatic cancer by various organizations are summarized in Table 2. 

## 4. How Often Should Patients Be Screened?

The surveillance interval for pancreatic cancer screening depends on the pancreatic tissue, the presence of cysts with or without worrisome features, the size of the solid lesion if present, and the stricture or dilatation (>6 mm) of the main pancreatic duct [77]. A cyst with a mural nodule, solid component, main pancreatic duct dilatation with an abrupt change in caliber, or thickened or enhanced cyst walls is considered a cyst with worrisome features that might require a closer follow-up or surgical resection [77]. Similarly, a solid lesion will require closer follow-up or surgical resection depending on the size of the lesion, dilatation of the duct, and endoscopic ultrasound fine needle aspiration [77]. There are no specific studies to determine the frequency of screening; however, the American Society of Gastrointestinal Endoscopy recommends annual screening for pancreatic cancer based on the model by Yu. et al. that assessed tumor growth based on mean differences in age between early and advanced tumors and the other model by Gangi et al. that analyzed imaging studies before the diagnosis of pancreatic cancer [78,80,81].

**Figure 1:**
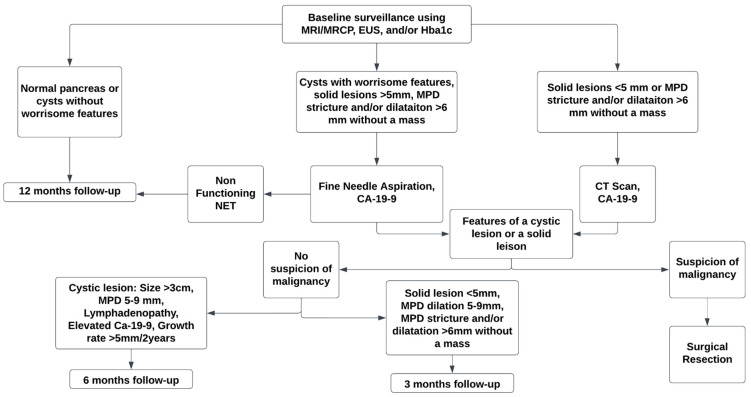
Outline for surveillance of pancreatic lesions in patients from high-risk groups (MRI: magnetic resonance imaging; MRCP: magnetic resonance cholangiopancreatography; MPD: main pancreatic duct; EUS: endoscopic ultrasound).

CAPS recommends annual screening in patients with normal pancreatic tissue or without concerning lesions and neuroendocrine tumors <10 mm, which aligns with the guidelines by the ASGE [77]. CAPS recommends a six-month follow-up in patients with cystic lesion of size ≥ 3 cm, a main pancreatic duct of 5–9 mm, lymphadenopathy, increased serum CA-19-9, and a growth rate of ≥5 mm/2 years [77]. A closer follow-up in 3 months is recommended for patients with solid lesions <5 mm, main pancreatic duct dilatation of 5–9 mm, and main pancreatic stricture and/or dilatation of ≥5 mm without a mass [77]. The AGA also has similar recommendations with a 6–12-month follow-up for low-risk lesions, 3–6 months for indeterminate lesions, and within 3 months for high-risk lesions [9,16]. A diagnostic approach for pancreatic cancer surveillance in patients from high-risk groups depending on the findings of initial testing is outlined in Figure 1. 

## 5. Modalities for Screening of Pancreatic Cancer

The goal of pancreatic cancer screening is to identify the premalignant lesions or resectable malignant lesions at an earlier stage in high-risk individuals to improve the overall survival rate [82,83]. An ideal screening protocol should be simple, affordable, and applicable to a generalized population that would help reduce mortality, avoid complications, and improve the patient’s quality of life [9]. Multiple modalities that can potentially be useful in the screening of pancreatic cancer are various genetic tests, biomarkers, as well as imaging techniques.

### Snapshotting Pancreatic Cancer: Exploring Early Detection with Imaging Techniques

At the moment, CT, MRI, and endoscopic ultrasonography (EUS) are the most often used imaging modalities for the pancreas. Numerous studies have assessed the precision of EUS, CT, and MRI for the identification of pancreatic primary tumors throughout the last 20 years. Functional imaging approaches are one of the innovative imaging modalities available for pancreatic cancer identification. Over the last two decades, screening for pancreatic cancer in high-risk individuals has been recommended with MRI or endoscopic ultrasound (EUS) depending on the expertise and experience of the providers [84]. Many studies have suggested the use of EUS for the better detection of small and solid lesions as compared to MRI for small cystic lesions [84]. A study conducted by Harnick et al. on 139 high-risk individuals showed that two solid tumors of <10 mm were detected by EUS only and 25% of cysts were detected by MRI [85]. A study conducted by Canto et al. comparing CT scan, MRI, and EUS in the evaluation of pancreatic lesions showed a sensitivity of 93% for EUS for solid lesions <2 cm as compared to 53% for CT scan and 67% for MRI [86]. Nine studies (*n* = 885) evaluated EUS-based screening with yields of pancreatic adenocarcinoma ranging from 0 (97.5% CI: 0.0–16.9) to 68.2 (95% CI: 14.3–186.6) cases per 1000 persons as compared to the yield of CT for pancreatic adenocarcinoma, which ranged from 0 (97.5% CI: 0.0–16.9) to 12.8 (95% CI: 0.3–69.4) per 1000 in 2 studies (n = 294) and the yield of pancreatic ductal adenocarcinoma ranging from 0 (97.5% CI: 0.0–16.9) to 75.0 (95% CI: 15.7–203.9) cases per 1000 persons across eight studies (n = 849). Numerous other studies have also shown that EUS has higher sensitivity (92–100%), specificity (89–100%), and accuracy (86–99%) in the detection of pancreatic malignancies, which is significantly higher than CT scan [87]. Endoscopic ultrasound fine needle aspiration can also be helpful in differentiating malignant from non-malignant masses as well to rule out alternate masses [87]. Data from four meta-analyses suggests that the sensitivity and specificity of EUS-FNA for the diagnosis of pancreatic cancer ranges from 85–92% and 96–98%, respectively [88,89,90,91].

The AGA recommends the use of EUS and MRI in combination for the screening of pancreatic cancer in high-risk individuals [16]. This aligns with the recommendation by the ASGE, and the modality of screening depends on the patient preference as well as available expertise [78]. The AGA also recommends the evaluation of low-risk lesions by EUS in 6–12 months, indeterminate lesions by EUS in 3–6 months, and the evaluation of high-risk lesions within 3 months [16]. The ASGE recommends EUS as the initial screening test in patients at very high risk for pancreatic cancer like Peutz–Jeghers syndrome and FAMMM as well as in patients for whom EUS can be combined with screening upper endoscopy or colonoscopy (e.g., Lynch and Peutz–Jeghers syndrome) [78]. The ASGE also recommends screening with linear EUS as compared to radial EUS due to the higher detection rate of pancreatic lesions with a linear echoendoscope based on a randomized controlled trial [92]. Contrast-enhanced MRI, preferably with a 3 T magnet for the better detection of soft tissue lesions, is recommended in patients at increased risk for adverse events during procedures or in patients who have a preference for non-invasive testing [78].

The advancement of pancreatic cancer diagnosis is greatly aided by nanomaterials and molecular imaging [93]. These technologies provide novel ways for early and accurate imaging evaluation of pancreatic cancer, in conjunction with procedures helped by artificial intelligence. Researchers hope to improve the prognosis for patients by improving the identification of pancreatic cancer, particularly in its early stages, by using targeted diagnostic magnetic nanoparticles and molecular imaging approaches. Furthermore, the combination of AI-assisted techniques with radiomics improves the accuracy and speed of pancreatic cancer diagnosis, which benefits patients [94].

## 6. Efficacy and Limitations of Current Screening Strategies for Pancreatic Cancer

Detecting pancreatic cancer in its early stages remains a difficult endeavor, owing to the absence of symptoms and the lack of specific biomarkers. Effective screening measures are critical, especially for high-risk populations, in order to increase early detection and perhaps improve patient outcomes. Thorough research was conducted to test the efficacy of numerous screening approaches for high-risk individuals. Endoscopic ultrasonography (EUS) and magnetic resonance imaging (MRI) are now the dominant procedures in this field. Endoscopic ultrasonography (EUS), in particular, has shown a high degree of sensitivity, revealing anomalies that would otherwise go undetected in conventional imaging exams. However, the accuracy of individual tests may vary, resulting in a range of good prediction findings. A thorough investigation revealed that the number of pancreatic adenocarcinoma cases diagnosed per 1000 people screened with EUS ranged from 0 to 68.2 [95]. A separate study highlighted the diagnostic accuracy of computed tomography (CT) in identifying pancreatic cancer, with a reported incidence of 0 to 12 cases per 1000 people [95,96]. Statistics show that screening for pancreatic cancer may identify cases; however, the efficiency of screening varies substantially depending on the individual studies and procedures utilized. These studies’ key findings highlight the potential of these methodologies, but they also highlight the need for further refinement in order to increase accuracy [95,96].

### 6.1. Challenges in Sensitivity and Specificity

The main challenge in pancreatic cancer screening is finding a balance between sensitivity and specificity. High sensitivity ensures that most malignant lesions are found, but it may also detect benign illnesses, resulting in false positives [95]. Conversely, high specificity may reduce false positives while increasing the chance of missing early tumors [96,97]. The detection and differentiation of small pancreatic lesions from benign illnesses remains a significant challenge. For example, the yield of first-time EUS in screening high-risk persons has been reported, with varying degrees of success in detecting true positives. The discovery of non-invasive, accurate biomarkers may revolutionize this aspect of screening, although such advancements are still on the horizon [97].

### 6.2. False-Positive Rates

False-positive results may have serious psychological and economic consequences [97,98]. Patients who receive false-positive results may experience increased anxiety, despair, and a perceived increase in their chances of developing cancer. These results also lead to increased healthcare use as patients undergo additional, often invasive, diagnostic testing to rule out cancer. The psychological impact of false positives, such as heightened cancer worries, has been documented, emphasizing the need for careful communication and follow-up [97]. The trade-off between the benefits of early detection and the risks associated with false positives is complicated and requires careful consideration.

### 6.3. Long-Term Outcomes 

The ultimate goal of any screening program is to improve long-term outcomes like survival rates and quality of life. Studies comparing the long-term outcomes of screened populations to unscreened cohorts have shown conflicting results [95,97]. While some studies suggest that screening may improve survival rates for high-risk individuals, the evidence is not conclusive. The long-term outcomes of screened vs. unscreened populations have been the focus of several studies. While some data indicate that screening may not significantly improve overall survival rates, concentrated screening in high-risk groups has demonstrated potential benefits [98]. The importance of early detection in improving survival rates, particularly for stage 0 and stage I cancers, has been highlighted by research findings [99]. The quality-of-life assessments, notably the psychological impact of screening, must also be addressed when evaluating these programs.

### 6.4. Psychological Impact

The psychological impact of screening on those at high risk for pancreatic cancer cannot be overstated. The technique may generate a wide range of emotions, from relief upon receiving a negative result to profound fear and despair if the results are positive or ambiguous. The perceived risk of developing cancer may also prompt significant lifestyle changes, some of which may not be necessary or beneficial. The psychological impact of screening is significant. Depending on the test results, studies have reported a wide range of emotional responses, including relief, fear, and despair [100]. These studies clearly demonstrate the need for psychological support and counseling for patients undergoing screening [100]. Healthcare practitioners must be equipped to deal with these psychological difficulties, providing guidance and support throughout the screening procedure. The efficacy and limitations of various imaging modalities are summarized in Table 3.

## 7. Promoter Methylation of ADAMTS1, BNC1, and Novel cfDNA Methylation Markers

The presence of cellular components in the circulation and other body fluids caused by malignant tumors and their metastases can open the door to early identification and new insights into diagnosis. Circulating tumor cells (CTCs), circulating cell-free nucleic acids (cfDNA and cfRNAs), and circulating extracellular vesicles called exosomes are some of these constituents. cfDNA is elevated in cancer patients in general [101] and particularly in pancreatic cancer [102]. cfDNA methylation analysis has emerged as a promising non-invasive approach for the identification of disease-specific signatures in pre-neoplastic lesions or for the early diagnosis of PC. The possibility of certain genes’ promoter methylation in cfDNA as early diagnostic indicators for pancreatic cancer has been brought to light by recent investigations. The cfDNA methylation status of two pancreatic-cancer-associated genes, *ADAMTS1* and *BNC1*, has been demonstrated to be highly sensitive and specific, enabling early pancreatic cancer diagnosis [103]. Furthermore, methylation promoter areas in the cfDNA of pancreatic cancer patients have been found for the genes *SPARC* (secreted protein acidic and rich in cysteine), *UCHL1* (ubiquitin carboxy terminal hydrolase L1), *PENK* (proenkephalin), and *NPTX2* (neuronal pentraxin 2). Interestingly, *SPARC* is an early marker for differentiating between PC and chronic pancreatitis, as demonstrated by Singh et al. [104]. Global alterations in cfDNA methylation patterns have been found in pancreatic cancer patients’ genome-wide epigenetic profiling assessment; 143 potential differentially methylated regions in promoter regions and CpG islands have been found [105]. Among these, methylation levels of MAPT, SIX3, MIR663, EPB41L3, FAM150A, TRIM73, LOC100128977, and LOC100130148 have been found to differ considerably between pancreatic cancer patients and healthy controls, suggesting that they may be useful as early diagnostic biomarkers [105]. Furthermore, a methylation-status-based diagnostic prediction model based on these eight indicators has been established, showing promising results in accurately differentiating between persons with pancreatic cancer and those without the condition [105]. These findings demonstrate the promise of cfDNA methylation analysis as a non-invasive method for the early identification of pancreatic cancer. In order to evaluate these markers and enable their easy adoption in clinical practice, more study is necessary. In the end, this will help patients with pancreatic cancer have a better prognosis. 

## 8. Exploring Novel Biomarkers for Pancreatic Cancer Detection

Due to late-stage detection, pancreatic cancer is an extremely aggressive cancer with a dismal prognosis. Improving patient outcomes and raising the likelihood of a successful course of treatment depend heavily on early identification. Finding new biomarkers for pancreatic cancer screening is essential to help in early detection and treatment.

Pancreatic cancer does not have a known early biochemical signature [106]. There is no proof that the traditional CA19-9 is an early event, despite the fact that it is typically elevated in pancreatic cancer. Other biochemical indicators that exhibit comparable behavior include osteopontin (as reviewed by Li et al. [107]), S600A6, and carcinoembryonic antigen (CEA). Most likely, one of the first signs is osteopontin [107]. Upon conducting an exhaustive review of the existing literature, the researchers uncovered no discernible early biochemical marker associated with pancreatic cancer [106].

The primary issue with the markers that are now available is not their sensitivity or specificity, but rather their “latency” in identifying the illness in its early stages [106]. The only serum biomarker approved by the FDA (United States Food and Drug Administration) for diagnostic purposes is CA19-9 (carbohydrate antigen 19-9) [108]. It was identified in 1979 in colorectal cancer and subsequently in pancreatic cancer [109,110]. Due to its incapacity to identify pancreatic cancers (PCs) without symptoms, the American Society of Clinical Oncology disapproves of the use of CA19-9 as a screening tool for the disease [111,112]. The fact that CA19-9 is elevated in benign conditions such as infection of the pancreas, liver, and gallbladder and has limited sensitivity and specificity for early-stage pancreatic cancer diagnosis, which reduces its diagnostic accuracy, supports this conclusion [113]. After screening more than 70,000 people between 1994 and 2000, Kim et al. [114] discovered that CA19-9 is inefficient in detecting groups that do not exhibit symptoms. CA19-9 can help in the evaluation of individuals with risk scores between 30 and 75 (as per a mathematical stimulation model developed [106]) when combined with diagnostic imaging. Although, it is crucial to remember that many patients with small pancreatic tumors may still not receive a diagnosis even when CA19-9 and imaging are used in tandem [115]. Another biomarker, osteopontin is a glycophosphoprotein that is released and produced by macrophages, infiltrates tumors, and is not a product of pancreatic tumor cells. In a study by Koopman et al. [116], osteopontin was shown to be elevated in the blood in all 50 patients with early diagnosis and surgical treatment, but not in any of the normal controls. Additionally, they demonstrated that elevated osteopontin had a 97% specificity and 80% sensitivity for pancreatic cancer. One issue with osteopontin is that it is elevated in chronic pancreatitis, making it difficult to distinguish between pancreatic inflammation and malignancy [117]. Although, this point has stirred up some controversies. According to Rychlikova et al. [118], pancreatic cancer can be diagnosed with an osteopontin level of more than 102 ng/mL. The problem stems from the fact that these kinds of high osteopontin levels are usually seen in later stages. In an investigation including 491 PC patients and 481 healthy individuals, it was discovered that early-stage pancreatic cancer patients had considerably higher osteopontin levels. However, whether any of the patients had a history of chronic pancreatitis was not made clear in the report [107].

TIMP-1s, also known as tissue inhibitor of metalloproteases-1, are a class of endogenous protein regulators that are involved in the matrix metalloproteinase (MMP) and ADAM families [119]. In pancreatic cancer, there is an overexpression of one of these inhibitors along with an increase in its serum level. Pancreatic cancer cells, particularly their stroma, exhibit significant levels of MMP2 (matrix metalloprotease 2) expression [120]. A kind of protein called MUC5AC is present in the pancreas and is subjected to glycosylation. It tends to be created in the early stages of abnormalities in pancreatic cells that might result in cancer and has a high molecular weight [121]. Glycoproteins, like MUC4, are big, heavy proteins that have been altered to include sugar molecules. While it is conspicuously missing in pancreatic inflammatory diseases, it is abundant in tissues afflicted by pancreatic cancer [122,123]. S600A6 is a protein that has been linked to the growth of tumors, and it has been found to be considerably overexpressed or upregulated in pancreatic cancer in particular [124]. In one study, Ohuchida et al. [125] measured the amounts of S600A6 mRNA in the pancreatic juice of both healthy people and people who had pancreatic cancer. They noticed that compared to patients without neoplastic conditions, those with pancreatic cancer, particularly those with chronic pancreatitis, had greater levels of S600A6. It is interesting to note that this increase in S600A6 was even seen early in the formation of pancreatic cancer, indicating its possible use as an early biomarker for the origin of pancreatic cancer. PC-594, a putative biomarker discovered by metabolomic analysis [126], shows significant decreases in pancreatic cancer patients as compared to healthy participants (0.76 ± 0.07 μmol/L versus 2.79 ± 0.15 μmol/L, respectively). Its potential value in pancreatic cancer diagnoses was supported by further study, which demonstrated a sensitivity of 90% and a specificity of 87% [127]. MIC-1, often referred to as macrophage inhibitory cytokine-1, was shown by Koopman et al. [128] to function as an early marker for differentiating between tumors that could be surgically removed and those that could not. MIC-1 has been identified as a separate biomarker from CA19-9, which is noteworthy. According to their research, MIC-1 performed better than CA19-9 in detecting pancreatic cancer patients when compared to healthy persons. However, its effectiveness in discriminating against pancreatic cancer from chronic pancreatitis was not as pronounced as per the study. In addition, hepatocyte growth factor (HGF), fibrinogen, and inflammatory markers such as C-reactive protein (CRP) and interleukin-6 (IL6) have been linked to pancreatic cancer instances. HGF has the potential to aid in the advancement of cancer but is not as useful in early diagnosis. It is highly raised in pancreatic cancer tissue samples and serum [129]. Despite being common in pancreatic cancer, inflammatory markers are not specific enough for early identification; instead, they work better as prognostic markers and to help differentiate pancreatic cancer from chronic pancreatitis [130]. Although fibrinogen levels, particularly the fibrinogen-to-albumin ratio, are thought to be more prognostic than diagnostic indicators, they do correlate with the development of tumors [131]. Due to sensitivity, specificity, and potential overlap with benign illnesses, a single biomarker may not be adequate for the early identification of pancreatic cancer. Enhancing early detection rates could be possible through the development of innovative techniques like metabolomic analysis or the combination of many indicators. Furthermore, more investigation is required to confirm these biomarkers’ effectiveness and practicality in clinical settings.

## 9. Unlocking the Potential of Circulating Exosomes: A Promising Avenue for Pancreatic Cancer Screening

Circulating exosomes have a number of advantages that make them a promising tool for pancreatic screening. These small vesicles are constantly being produced by both normal and tumor cells, although tumor cells produce them at a higher rate. The contents of circulating exosomes often contain diagnostic signals for pancreatic cancer, which may provide a non-invasive approach to the early detection of the pathology through blood sampling. Tumor-derived exosomes were vividly portrayed as a “message in a bottle” by Kharaziha et al. [132]. Exosomes’ diagnostic utility is enhanced by their ability to distinguish cancer from benign diseases, such as inflammatory pancreatic disorders, and to provide diagnostic signals specific for pancreatic cancer. Exosomes often reveal the cancer’s genetic markers, which further improves diagnostic accuracy. Experimental data show that circulating exosomes are highly sensitive to pancreatic cancer, with a sensitivity of more than 100% [133]. This sensitivity is partly due to the increased production of these substances in malignant cells, further increasing their diagnostic usefulness [134]. Within body fluids, exosomes are rather stable. Harvesting them is therefore not a difficult task [135]. The upregulation of miRNAs-21 and -221 in tissues from pancreatic cancer emphasizes how crucial exosome content is for diagnosis. The aforementioned miRNAs highlight the potential of exosome analysis in early diagnosis and therapy monitoring.

## 10. Advancing Pancreatic Cancer Screening: Future Directions and Recommendations

The stealthy progression of pancreatic cancer makes it one of the most fatal kinds of cancer, emphasizing the critical need for effective screening tools, particularly for those at high risk. This article discusses anticipated improvements in screening methods, the use of cutting-edge technology, and the significance of individualized risk assessment, all with the objective of boosting early detection rates and patient outcomes.

### 10.1. Integration of Multi-Modal Approaches

Pancreatic cancer screening is currently being implemented using a variety of techniques. We may increase diagnostic accuracy by combining traditional imaging modalities such as CT scans and MRIs with contemporary endoscopic procedures such as endoscopic ultrasonography (EUS), and then evaluating the data with biomarker profiles such as CA-19-9 levels [136,137]. This comprehensive technique aims to give a full assessment of the pancreas, perhaps uncovering abnormalities that would otherwise go undiscovered if just one screening method were used.

### 10.2. Advancements in Technology

Technological advances are critical to the future of pancreatic cancer screening. The combination of modern imaging technologies and the application of artificial intelligence to image processing has the potential to totally revolutionize detection capabilities [86]. Furthermore, modern approaches to genomes, proteomics, and metabolomics enable the development of novel biomarkers. This has the potential to enhance screening procedures and identify cancer in its early stages [138].

### 10.3. Personalized Risk Assessment

The trend toward personalized treatment is becoming increasingly apparent in cancer-screening initiatives. By incorporating genetic profiling, environmental exposures, and individual health histories into risk assessment models, screening recommendations may be tailored to each patient’s distinct risk profile [86,136]. This tailored technique not only improves the expected accuracy of screening programs, but it also eliminates unnecessary treatments for people who are thought to be at a lower risk [16,138].

### 10.4. Interdisciplinary Collaboration

The complex nature of pancreatic cancer emphasizes the need for interdisciplinary collaboration across different medical and scientific fields. Gastroenterologists, oncologists, radiologists, geneticists, and epidemiologists must collaborate to create screening tools, interpret complex data, and use findings in clinical practice. Such collaborative efforts are critical for constantly developing screening approaches and improving patient outcomes.

### 10.5. Prospective Studies

Evidence-based medicine is built on robust, prospective research. Such trials are critical in determining the real-world efficacy and cost-effectiveness of various pancreatic cancer-screening approaches [16]. Researchers may gain critical insights into the long-term benefits and potential drawbacks of screening methods by meticulously tracking outcomes over time, which will impact policy formulation and clinical decision-making.

The advancement of pancreatic cancer screening is based on a multifaceted approach that includes the use of cutting-edge technologies, individualized risk assessments, and collaborative research. Prospective studies will serve as compasses, tracing the course of various procedures while ensuring their efficacy and efficiency. By implementing these recommendations, the medical community may make significant progress in the early detection of pancreatic cancer, providing new hope for improved patient survival and quality of life. A summary of different strategies for the early detection of pancreatic cancer is presented in Table 4.

## 11. Conclusions

The identification and management of precursor lesions and the early stages of pancreatic cancer is crucial as advanced pancreatic cancer is associated with lower survival rates. Based on the guidelines by various organizations, screening for pancreatic cancer should be avoided in asymptomatic, average-risk individuals. However, screening for pancreatic cancer is favorable in high-risk populations who carry germline mutations associated with pancreatic cancer and have recently experienced the onset of diabetes mellitus. The preferred imaging modality for pancreatic cancer surveillance depends on the lesion characteristic during baseline screening, as well as provider expertise. There is significant advancement in developing novel biomarkers to detect pancreatic cancer in the early stages; however, there is no specific marker identified yet to diagnose early pancreatic cancer. It is also important for clinicians to consider the long-term and socioeconomic impact of pancreatic cancer surveillance on individuals, and optimal strategies should be adapted to improve outcomes and minimize harms. Further studies are needed to fully identify and define the high-risk populations who should be screened for pancreatic cancer, as well as the optimal protocol for better patient outcomes.

## Figures and Tables

**Table 1:** Risk of Pancreatic Cancer Associated with Various Hereditary Conditions.

Genetic Syndrome	Genes Affected	Risk of Pancreatic Cancer
Peutz–Jeghers Syndrome	*STK11*	Relative Risk: 132; Cumulative Risk: 2.4% at age 40, 3.9% at age 50, 11.1% at age 60, and 25.6% at age 70 years
Lynch Syndrome	*MLH1, MSH2, MSH6, PMS2*	Relative Risk: 5–9; Cumulative Risk: 1.3% by age 50 years, 3.7% by age 70 years
Hereditary Breast and Ovarian Cancer	*BRCA1, BRCA2, PALB2*	BRCA1: Relative Risk: 2.26; BRCA2: Relative Risk: 3.51
Familial Atypical Multiple Mole Melanoma	*CDKN2A*	Cumulative Risk: 17% by age 75 years
Li–Fraumeni Syndrome	*TP53*	Relative Risk: 7.73
Familial Adenomatous Polyposis	*APC*	Hazard Ratio: 6.45; Relative Risk: 4.46
Ataxia–Telangiectasia	*ATM*	Relative Risk: 6.5
Cystic Fibrosis	*CFTR*	Risk Ratio: 5.3 (95% CI: 2.4–10.1)
Hereditary Pancreatitis	*PRSS1*	Standardized Incidence Ratio: 53 (95% CI: 23–105) Cumulative Risk: 40% by age 70 years

**Table 2:** Recommendations for Pancreatic Cancer Screening in High-Risk Groups.

Risk Group	AGA Recommendation	ASGE Recommendation	CAPS Recommendation
Familial Pancreatic Cancer	Start screening at age 50 or 10 years younger than the initial age of onset	Start screening at age 50 or 10 years younger than the youngest relative	Start screening at age 50 or 10 years younger than the youngest relative
Peutz–Jeghers Syndrome	Start screening at age 35	Start screening at age 35	Start screening at age 40 or 10 years younger than youngest relative (Grade 2: probably do it); Start screening at age 30 or 35 (Grade 4: probably do not do it)
*PRSS1* Mutation	Start screening at age 40	Start screening at age 40	Start screening at age 40
*CDKN2A* Mutation	Start screening at age 40	Start screening at age 40	Start screening at age 40
*BRCA2* Mutation	Start screening at age 50 or 5 years earlier than high-risk individuals	Start screening at age 50 or 10 years younger than the youngest relative	Start screening at age 50 or 5 years earlier than high-risk individuals
*ATM* Mutation	Start screening at age 50	Start screening at age 50 or 10 years younger than the youngest relative	Start screening at age 50
*PALB2* Mutation	Start screening at age 50 or 5 years earlier than high-risk individuals	Start screening at age 50 or 10 years younger than the youngest relative	Start screening at age 50 or 5 years earlier than high-risk individuals
Lynch Syndrome	Start screening at age 50 or 10 years younger than the youngest relative	Start screening at age 50 or 10 years younger than the youngest relative	Not specified
*TP53* Mutation	Start screening at age 50 or younger depending on family history	Not specified	Not specified

**Table 3:** Pancreatic Cancer Screening: A Balance of Efficacy and Challenges.

Aspect	Efficacy	Limitations
Screening Modalities	EUS and MRI are primary methods, with EUS showing high sensitivity in detecting anomalies.	Accuracy varies, and no single method is universally effective.
Diagnostic Yield	EUS can detect 0 to 68.2 cases of pancreatic adenocarcinoma per 1000 individuals screened.	The yield for CT is lower, with 0 to 12 cases per 1000 individuals.
Sensitivity and Specificity	High sensitivity of screening methods ensures most malignant lesions are detected.	High sensitivity may also detect benign conditions, leading to false positives.
False-Positive Rates	-	False positives can cause psychological distress and unnecessary healthcare utilization.
Long-Term Outcomes	Some studies suggest potential survival benefits for high-risk individuals through screening.	Conflicting results on whether screening significantly improves overall survival rates.

**Table 4:** Comprehensive Strategies in Pancreatic Cancer Screening: A Multi-Modal Approach.

Multi-Modal Approach	Description
Imaging Techniques	Combining CT scans, MRIs, and endoscopic ultrasound (EUS) to provide a comprehensive view of the pancreas and detect lesions.
Biomarker Analysis	Utilizing blood tests for biomarkers like CA 19-9 alongside imaging to improve the detection of pancreatic cancer.
Genetic Screening	Incorporating genetic testing for hereditary cancer syndromes that increase the risk of pancreatic cancer.
Artificial Intelligence	Applying AI algorithms to analyze imaging and biomarker data for enhanced accuracy in diagnosis.
Interdisciplinary Evaluation	A collaborative approach where gastroenterologists, oncologists, radiologists, and geneticists work together to interpret screening results.
